# Sample Size Guidelines for Logistic Regression from Observational Studies with Large Population: Emphasis on the Accuracy Between Statistics and Parameters Based on Real Life Clinical Data

**DOI:** 10.21315/mjms2018.25.4.12

**Published:** 2018-08-30

**Authors:** Mohamad Adam Bujang, Nadiah Sa’at, Tg Mohd Ikhwan Tg Abu Bakar Sidik, Lim Chien Joo

**Affiliations:** 1 Clinical Research Centre, Sarawak General Hospital, Ministry of Health, Kuching, Malaysia; 2 Biostatistics Unit, National Clinical Research Centre, Ministry of Health, Kuala Lumpur, Malaysia

**Keywords:** logistic regression, observational studies, sample size

## Abstract

**Background:**

Different study designs and population size may require different sample size for logistic regression. This study aims to propose sample size guidelines for logistic regression based on observational studies with large population.

**Methods:**

We estimated the minimum sample size required based on evaluation from real clinical data to evaluate the accuracy between statistics derived and the actual parameters. Nagelkerke r-squared and coefficients derived were compared with their respective parameters.

**Results:**

With a minimum sample size of 500, results showed that the differences between the sample estimates and the population was sufficiently small. Based on an audit from a medium size of population, the differences were within ± 0.5 for coefficients and ± 0.02 for Nagelkerke *r*-squared. Meanwhile for large population, the differences are within ± 1.0 for coefficients and ± 0.02 for Nagelkerke *r*-squared.

**Conclusions:**

For observational studies with large population size that involve logistic regression in the analysis, taking a minimum sample size of 500 is necessary to derive the statistics that represent the parameters. The other recommended rules of thumb are EPV of 50 and formula; *n* = 100 + 50*i* where *i* refers to number of independent variables in the final model.

## Introduction

Logistic regression is one of the most utilised statistical analyses in multivariable models especially in medical research. Beside the fact that most clinical outcomes are defined as binary form (e.g. survived versus died or poor outcome versus good outcome), logistic regression also requires less assumptions as compared to multiple linear regression or Analysis of Covariance (ANCOVA). In observational studies, logistic regression is commonly used to determine the associated factors with or without controlling for specific variables and also for predictive modelling (1–4). Since the purpose of most of statistical analyses is for inference, determination of sample size requirement is necessary before the analysis is conducted.

The sample size requirement for logistic regression has been discussed in the literature. Earlier on, Hsieh (5) proposed a sample size table for logistic regression but limited the estimation for only one covariate. According to the paper, adjustment needed to be made for the sample size tables such as dividing the estimated sample size with a factor of (1–*p*^2^) when sample size need to be estimated for logistic regression. Another famous sample size guideline proposed that the minimum required sample size should be based on the rule of event per variable (EPV) (6). According to Concato et al. and Peduzzi et al., the concept of EPV of 10 is acceptable for both logistic regression and cox regression (6–7).

Based on EPV, researchers need to estimate the proportion for the outcome in the least category and divide it by 10 in order to determine the number of independent variables which can be studied. The concept of EPV with 10 received some critics (8) and hence, Austin and Steyerberg recommended EPV of 20 instead (9). Besides that, studies with small to moderate samples size such as less than 100 usually overestimate the effect measure. Nemes and colleagues from their simulation study, showed that large sample size preferably 500 will increase the accuracy of the estimates (10). The rules of thumb with 500 subjects were also been recommended by other studies (11–12). In these studies, sample size with 500 and above yielded statistics which represented the parameters in the targeted population. The results were derived after evaluating few populations and were analysed based on various statistical tests.

The present study did an evaluation using real patient data derived from an observational study to evaluate the extent of different sample sizes used in affecting the discrepancy between the sample statistics and the actual parameters in the target population. The purpose of this comparison is to estimate a minimum sample size required for a research study which is able to yield the closest estimate for the coefficients and also *r*-squared. This is to determine a sizeable sample size for logistic regression that can produce the statistics which is able to be inferred to the larger population particularly for observational studies.

Sample size for experimental studies are usually calculated using sample size softwares. In experimental studies, the confounders are usually controlled at study design stage and this made the calculation is feasible based on univariate analysis. The researcher only need to estimate of effect sizes in order to calculate the minimum requirement of sample size. Very often, observational studies will involve multivariable analysis with many parameters and various effect sizes. Therefore, in the present study, we propose a simple rule of thumb as a basis for sample size estimation for logistic regression particularly for observational studies. In the perspective of observational studies, the findings obtained from the validation of real data were used as the basis for sample size recommendation for logistic regression.

## Material and Methods

Validation was conducted to verify the accuracy between statistics and parameters. The validation was performed using real patient data from “An Audit of Diabetes Control and Management (ADCM) 2009”, which included all data collection (at a national-level) of patients with diabetes mellitus from all government health clinics in Malaysia in 2009. The methodology of this data collection process was explained in a previous paper and published elsewhere (13). We selected one government health clinic which had a relatively high number of patients with a total population of 1,595, and re-analysis was done by using different sub-samples (*n* = 30, 50, 100, 150, 200, 300, 500, 700 and 1,000).

We tested a multivariable model by using eight explanatory (or independent) variables and one outcome (or dependent variable). The dependent variable was glycemic control (HbA1c) in binary form (< 7.0 versus ≥ 7.0) while a set of independent variables included gender, age, body mass index, diabetes treatment, duration of diabetes mellitus, systolic blood pressure, status of co-morbidity and low-density lipoprotein level. Since data was not collected in a prospective manner, the model developed could only be used to test for an association between the independent variables and the outcome; rather than to identify and determine the risk factors or determinants for HbA1c (14–15).

The findings obtained from the validation were then analysed. The statistics such as *r*-squared and coefficients derived from the samples were compared with the respective true values (parameters) in the targeted population. The analysis was conducted using logistic regression where the sample sizes (*n* = 30, 50, 100, 150, 200, 300, 500, 700 and 1,000) were selected at random. From the results, guidelines of sample size estimation for logistic regression based on the concept of event per variable (EPV) and sample size formula (*n* = 100 + *xi*, where *x* is integer and *i* represents the number of independent variables in the final model) were introduced.

After the guidelines of the sample size were identified, these guidelines (based on EPV and sample size formula) were re-evaluated based on another extremely large population with total population of 70,899 records. This population was also from ADCM 2009 registry but included all notification records from participating health clinics in 2009. The approach in the analysis of the logistic regression model is similar to the approach of analysis as presented in Table 1. Existing rules of thumb for sample size using logistic regression are highly dependent on the number of independent variables. Therefore, the evaluation using very large population is necessary to determine whether these guidelines can still provide satisfactory results (results yield minimal bias between results derived from parameters and statistics, respectively).

For data management, single imputation technique was applied to replace the missing values where the missing in numerical values were replaced with mean and missing in categorical values were replaced with mode. The logistic regression was conducted without stepwise method (enter method). All the analyses were carried out using IBM SPSS version 21.0 (IBM Corp. Released 2012. IBM SPSS Statistics for Windows, Version 21.0. Armonk, NY: IBM Corp.).

## Results

### Validation

**Figure 1:**
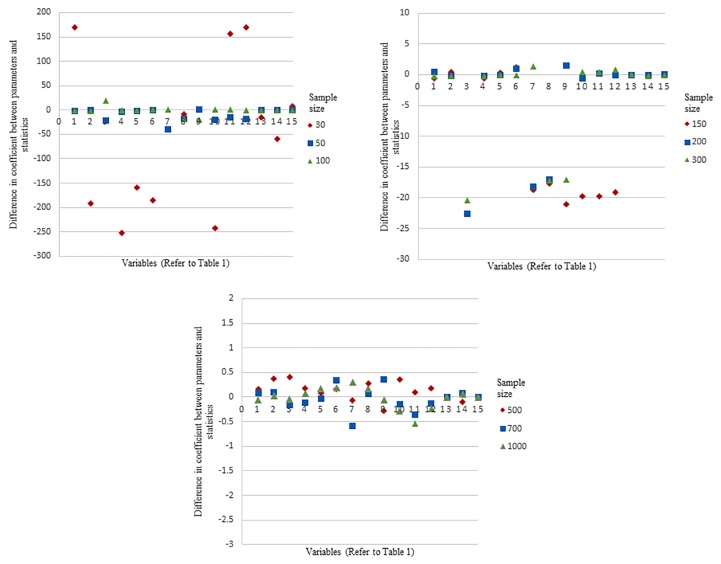
The comparison of differences of coefficients between results derived from parameters and statistics based on various sample sizes

**Figure 2:**
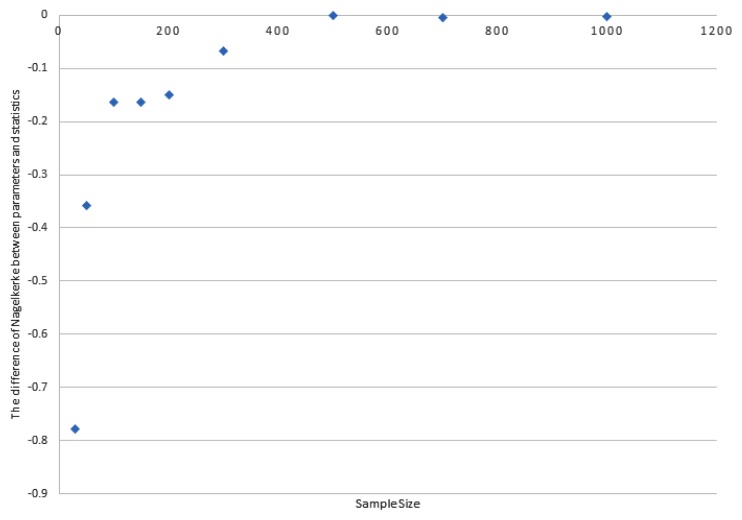
The comparison of differences of Nagelkerke *r*-squared between results derived from parameters and statistics based on various sample sizes

The details of the variables are presented in Table 1 and results obtained from the validation are illustrated in Figure 1 and Figure 2. The validation involved eight independent variables with five categorical variables and three numerical variables. Based on Figure 1 and Figure 2, results showed that with a minimum sample size of 500, it is possible to ensure that the differences between the sample estimates and the population parameters such as regression coefficients and Nagelkerke *r*-squared to be sufficiently small (i.e. differences within ± 0.5 for coefficients and differences within ± 0.02 for Nagelkerke *r*-squared). This indicates that a minimum sample size of 500 will yield reliable and valid sample estimates for the targeted population.

### Comparison with the Approach Based on EPV and Formula; n = 100 + xi

Previous studies introduced a minimum guideline for EPV (6). These guidelines were re-evaluated based on a real-life clinical data with emphasis on the accuracy between statistics and sample. The parameter of poor control of HbA1c level was known with 80.0%. When taking a rule of thumb with EPV of 10, sample size of 100 is sufficient for eight independent variables. However, results based on the validation for sample size of 100 yielded a lot of bias in the coefficients and Nagelkerke *r*-squared. The findings showed that statistics which could represent the true values in the population could only be achieved with EPV of 50 (Table 2).

A simple formula such as *n* = 100 + *xi* (*x* is integer and *i* represents number of independent variable in the final model) was introduced as a basis of sample size for logistic regression particularly for observational studies where the sample size emphasised the accuracy of the statistics. The recommended rule of thumb was *n* = 100 + 50(*i*) in which this formula would yield 500 subjects since *i* was equivalent to eight (independent variables) (Table 2).

### Re-Evaluation of the Rules of Thumb

**Figure 3:**
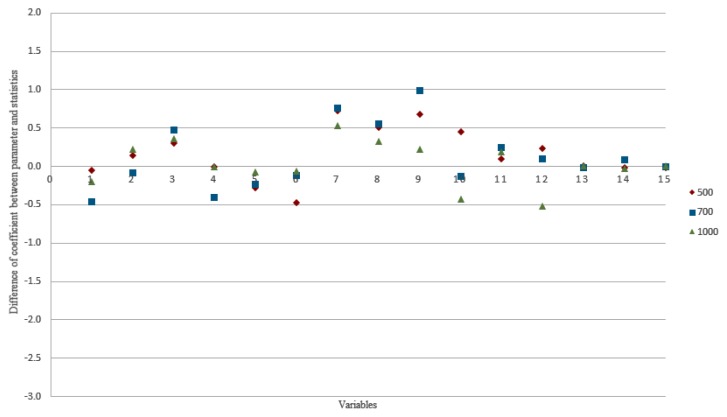
The comparison of differences of coefficients between results derived from parameters and statistics based on various sample sizes tested with larger sample

The rules of thumb based on EPV 50 and *n* = 100 + 50(*i*) were selected. The sample size based on these rules of thumb were re-evaluated in another different and extremely large population. The analysis yielded minimum bias in terms of coefficient (comparing between coefficients from the parameter and the respective statistics) based on sample size 500 and more (Figure 3). This indicated the suitability of sample size based on EPV 50 and formula *n* = 100 + 50(*i*) which were not affected by the total number of the population. The difference in Nagelkerke *r*-squared between parameter and statistics of 500 subjects, 700 subjects and 1,000 subjects were −0.013, −0.016 and −0.014, respectively. The differences in coefficients between parameter and statistics of 500 subjects, 700 subjects and 1,000 subjects ranged between −0.457 and 0.986.

## Discussion

Conventionally, the minimum required sample size for almost all types of multivariable analysis is determined using a rule-of-thumb such as for MLR/ANCOVA (16–17), logistic regression (5–6) and exploratory factor analysis (18–20). This is because multivariable analysis involves many parameters and those parameters are sometimes difficult to estimate. In this study, we proposed a simple guideline to determine sufficient sample size for logistic regression particularly for observational studies in large population. The emphasis is to estimate sizeable effect size that is able to derive the closest estimates for the parameters in the targeted population.

Based on the findings, sample size with at least 500 is able to produce statistics that are nearly representative of the true values in the targeted population. This recommended sample size of 500 had also been proposed in previous studies (11–12). The present study proposes a desirable sample size to detect a close approximation for the parameters in the targeted population and the aim is to be able to detect an almost accurate for low to large effect sizes.

A major concern of performing a statistical analysis is the validity of the inference drawn from the results obtained from a sample, and whether such inference can be a close approximation of the true value obtained from the target population. In other words, either low, medium or large effect sizes found in an inferential analysis might not represent the true effect size for the targeted population. The only way to know this is by conducting census study which challenging and costly.

In any research study that involves inferential analysis, there is a possibility that the research findings is false (21). This is because, most inferential studies rely on the *P*-value less than 0.05 or 0.01 as the indicator of evidence for inference where the parameters remain unknown until census study is conducted for a particular population (22–23). Therefore, to ensure the estimates are valid, it is recommended that research studied to be conducted with a sufficient sample size especially when the analysis involves multivariable analysis and this is usually the case for observational studies (11–12, 24–27).

The present study introduces a simpler formula for sample size estimation particularly for logistic regression in observational studies. This study proposes a formula of *n* = 100 + *xi* where *x* is any integer and *i* represents number of independent variable. The basis of the formula is that sample size is determined by two factors which are an integer and number of independent variables. The constant of 100 is fixed based on a previous a study which reported that a sample size of 100 or less for logistic regression is not sufficient (10). In this study, *i* is fixed at eight and thus an appropriate integer needed to be determined next. Based on the validation result, the reasonable value for *x* is 50. Therefore, for eight independent variables, the sufficient sample size to be able to derive statistics that is presentative of the parameter in the targeted population is 500 (500 = 100 + [50 × 8]). Hence, based on the concept of EPV, the recommended rule of thumb is EPV is 50.

In sample size estimation, it is well understood that a smaller sample size is needed to detect large effect size. In other words, sample size lower than 500 is sufficient if the aim of the analysis is to determine factors which are highly associated with an outcome. However, the common problem in research is that the effect size is unknown most of the times. Hence, to purposely estimate a lower sample size with the assumption that the estimated effect sizes are large can introduce bias. To overcome the problem, researchers need to be able to estimate an almost accurate effect sizes based on literatures. Besides that, majority of multivariable analysis such as logistic regression will involve stepwise analysis, resulting in only independent variables with large effect size to be remained in the result (1–2). Therefore, a lower rule of thumb such as EPV of 10 and 20 are still relevant and this subject to in a case for medium to large effect size.

For observational studies with large population that involves logistic regression analysis, a minimum sample size of 500 is necessary to derive the statistics that represent the parameters in the targeted population. The other recommended rules of thumb are EPV of 50 or formula of *n* = 100 + 50(*i*) where *i* refers to number of independent variables in the final model. Sample size less than 500 or sample size derived from EPV of 50 or *n* = 100 + 50*i* could also be sufficient provided the result from the analysis yields medium to large effect sizes.

These rules of thumb have been tested in extremely large population in this study and results showed that the statistics derived from the sample were almost similar to the parameters in the population. The possibility in getting the minimum bias when sample size of 500 was used could be potentially happen because the statistics were derived based on almost 30% of the total population (30/100 × 1595 = 478.5). However, the same rules of thumb are still able to provide minimum bias after testing the same logistic regression model in large population (*N* = 70,899). This indicates that the suitability of sample size based on EPV 50 and formula *n* = 100 + 50(*i*) is not directly influenced by the total number of the population.

Previous study by Hsieh et al. (28) proposed a formula to estimate sample size for multivariable logistic regression based on desired effect sizes such as odd ratio and *r*-squared. The major difference between the study by Hsieh et al. (28) and present study is the basis of sample size determination in which Hsieh et al. (28) used the formula based on the statistical test of logistic regression to determine the sample size while present study proposed the rule of thumb based on an audit or validation from population data. The concept proposed by Hsieh et al. (28) is more suitable for experimental studies as sample size estimation is based on the effect size. However, to determine the effect sizes for observational studies such as studies to determine the associated factors toward an outcome can be difficult since the analysis involves multiple variables. Therefore, the present study proposed a simpler rule of thumb to estimate sample size for non-experimental studies.

One of the limitations of this study is that the validation was tested based on a single dataset. However, previous studies tested various datasets and the findings were consistent with the present study (11–12). The other limitation is that simulation analysis was not conducted due to a few reasons. Sample size guideline based on simulation is dependent on the model setting and it is understood that there are various regression models that can be developed since the models can involve small to large number of independent variables and various pre-specified effect sizes can be allocated for the simulation purpose.

Therefore, various types of simulation with different models can be difficult to be conducted in a single paper. In this present study, the parameters are already known, hence it is feasible to compare the bias between statistics and parameters based on each sub sample taken by random. To test the robustness of the results, validation based on various real-life datasets are necessary for recommendation in future studies. Sample size guidelines based on simulation analysis have been conducted in other studies (6, 10) with different models. Study by Nemes et al. recommended sample size of 500 which is a similar recommendation in this present study (6).

## Conclusions

In conclusion, for observational studies that involve logistic regression in the analysis, this study recommends a minimum sample size of 500 to derive statistics that can represent the parameters in the targeted population. The other recommended rules of thumb are EPV of 50 and formula; *n* = 100 + 50*i* where *i* refers to number of independent variables in the final model. However, sample size less than 500 may be sufficient for associations that yield medium to large effect size.

## Figures and Tables

**Table 1:** Information for an audit data, variables name and the code

Variables	Code for variable
**Outcome**	
HbA1c	
Poor	Reference group
Good	1
**Associated factors**	
*Categorical form*	
Gender	
Male	1
Female	Reference group
BMI Category	
Normal	2
Underweight	3
Overweight	4
Obese	Reference group
Duration of diabetes	
< 5 years	5
5–10 years	6
> 10 years	Reference group
Treatment	
Diet only	7
Oral ADA only	8
Insulin only	9
Both oral and insulin	Reference group
Co-morbidity	
No	Reference group
Hypertension only	10
Dyslipidemia only	11
Hypertension and Dyslipidemia	12
*Numerical form*	
Age	13
Low-density lipoprotein	14
Blood pressure (systolic)	15

**Table 2:** Comparison with the basis of sample size based on rule of thumb between EPV (prevalence of poor control = 80.0% and number of independent variables = 8) and formula of *n* = 100 + *xi* (*x* is integer and *i* represents number of independent variable)

Guideline	Minimum sample in poor control	Minimum sample size based on EPV	Number of independent variables	Minimum sample size based on formula
*EPV*				
EPV of 10	80	100 (80 in poor outcome category)		
EPV of 20	160	200 (160 in poor outcome category)		
EPV of 30	240	300 (240 in poor outcome category)		
EPV of 40	320	400 (320 in poor outcome category)		
EPV of 50	400	500 (400 in poor outcome category)		
*Formula*				
100 + 10 (*i*)			8	180
100 + 20 (*i*)			8	260
100 + 30 (*i*)			8	340
100 + 40 (*i*)			8	420
100 + 50 (*i*)			8	500

## References

[1] Chew BH, Shariff-Ghazali S, Mastura I, Haniff J, Bujang MA (2013). Age ≥ 60 years was an independent risk factor for diabetes-related complications despite good control of cardiovascular risk factors in patients with type 2 diabetes mellitus. Exp Gerontol.

[2] Chew BH, Mastura I, Shariff-Ghazali S, Lee PY, Cheong AT, Ahmad Z (2012). Determinants of uncontrolled hypertension in adult type 2 diabetes mellitus: an analysis of the Malaysian diabetes registry 2009. Cardiovasc Diabetol.

[3] Lee PY, Cheong AT, Zaiton A (2013). Does ethnicity contribute to the control of cardiovascular risk factors among patients with type 2 diabetes?. Asia Pac J Public Health.

[4] Premsenthil M, Salowi MA, Bujang MA, Kueh A, Siew CM, Sumugam K (2015). Risk factors and prediction models for retinopathy of prematurity. Malays J Med Sci.

[5] Hsieh FY (1989). Sample size tables for logistic regression. Stat Med.

[6] Peduzzi P, Concato J, Kemper E, Holford TR, Feinstein AR (1996). A simulation study of the number of events per variable in logistic regression analysis. J Clin Epidemiol.

[7] Concato J, Peduzzi P, Holford TR, Feinstein AR (1995). The importance of event per variable (EPV) in proportional hazard analysis: I. Background, goals and general strategy. J Clin Epidemiol.

[8] van Smeden Maarten, de Groot JAH, Moons KGM, Collins GS, Altman DG, Eijkemans MJC (2016). No rationale for 1 variable per 10 events criterion for binary logistic regression analysis. BMC Med Res Methodol.

[9] Austin PC, Steyerberg EW (2017). Events per variable (EPV) and the relative performance of different strategies for estimating the out-of-sample validity of logistic regression models. Stat Methods Med Res.

[10] Nemes S, Jonasson JM, Genell A, Steineck G (2009). Bias in odds ratios by logistic regression modelling and sample size. BMC Med Res Methodol.

[11] Bujang MA, Ghani PA, Zolkepali NA, Selvarajah S, Haniff J (2009). A comparison between convenience sampling versus systematic sampling in getting the true parameter in a population: explore from a clinical database: the Audit Diabetes Control Management (ADCM) registry in 2009. Int Conf Stat Sci Bus Eng.

[12] Bujang MA, Sa’at N, Joys AR, Ali MM (2015). An audit of the statistics and the comparison with the parameter in the population.

[13] Mastura I, Chew BH, Lee PY, Cheong AT, Sazlina SG, Jamaiyah H (2011). Control and treatment profiles of 70,889 adult type 2 diabetes mellitus patients in Malaysia. International Journal of Collaborative Research on Internal Medicine & Public Health.

[14] Cheong AT, Lee PY, Sazlina S-G, Bujang MA, Chew BH, Mastura I (2013). Poor glycemic control in younger women attending Malaysian public primary care clinics: findings from adults diabetes control and management registry. BMC Fam Pract.

[15] Khattab M, Khader YS, Al-Khawaldeh A, Ajlouni K (2010). Factors associated with poor glycemic control among patients with type 2 diabetes. J Diabetes Complications.

[16] Tabachnick BG, Fidell LS (2013). Using multivariable statistics.

[17] Bujang MA, Sa’at N, Tg Abu Bakar Sidik TMI (2017). Requirement for multiple linear regression and analysis of covariance based on experimental and non-experimental studies. Epidemiology Biostatistics and Public Health.

[18] MacCallum RC, Widaman KF, Zhang S, Hong SH (1999). Sample size in factor analysis. Psychol Methods.

[19] Osborne JW, Costello AB (2004). Sample size and subject to item ratio in principal components analysis. Pract Assess Res Eval.

[20] Bujang MA, Ghani PA, Soelar SA, Zulkifli NA (2012). Sample size guideline for exploratory factor analysis when using small sample: taking into considerations of different measurement scales.

[21] Ioannidis JPA (2005). Why most published research findings are false. PLoS Med.

[22] Sterne JA, Davey SG (2001). Sifting the evidence— what’s wrong with significance tests. BMJ.

[23] Wacholder S, Chanock S, Garcia-Closas M, Elghormli L, Rothman N (2004). Assessing the probability that a positive report is false: an approach for molecular epidemiology studies. J Natl Cancer Inst.

[24] Sedlmeier P, Gigerenzer G (1989). Do studies of statistical power have an effect on the power of studies?. Psychol Bull.

[25] Rossi JC (1990). Statistical power of psychological research: what have we gained in 20 years?. J Consult Clin Psychol.

[26] Muller KE, Benignus VA (1992). Increasing scientific power with statistical power. Neurotoxicol Teratol.

[27] Cohen J (1994). The earth is round (*P* < .05). Am Psychol.

[28] Hsieh FY, Bloch DA, Larsen MD (1998). A simple method of sample size calculation for linear and logistic regression. Statist Med.

